# Lumbar Pedicle Morphometry of Dry Vertebral Columns in Relation to Transpedicular Fixation: A Cross-Sectional Study From Central India

**DOI:** 10.7759/cureus.38108

**Published:** 2023-04-25

**Authors:** Virendra Verma, Udit Agrawal

**Affiliations:** 1 Orthopedics, All India Institute of Medical Sciences, Bhopal, IND

**Keywords:** vertebrae, pedicle screw, lumbar, anatomy, pedicle

## Abstract

Introduction: The lumbar vertebrae are the largest vertebrae of the vertebral column, which support the maximum body weight. There has been an increased focus on transpedicular spinal fixation for addressing various lumbar spine pathology. However, its safety and efficacy require precise knowledge of the lumbar pedicle anatomy. Mismatched size of screw and pedicle may lead to failure of instrumentation. It may result in cortex perforation or pedicle fracture and loosening of the pedicle screw. The oversizing of the pedicle screw can result in dural tears, leakage of the cerebrospinal fluid, and injuries to the nerve root. As the racial variations in the anatomy of a pedicle are well known, this study was performed to assess the morphological parameters of the lumbar vertebrae pedicles in the Central Indian population so that the appropriate sizes of pedicular implants can be selected.

Material and methods: The present study was conducted at a tertiary-level hospital and medical college on dry lumbar vertebrae specimens available in the department of anatomy. The measurement of morphometric parameters of the lumbar vertebrae pedicles was performed in 20 dry lumbar specimens using vernier calipers and a standard goniometer. The morphometric parameters included in the study are pedicle transverse external diameter (pedicle width), pedicle sagittal external diameter (pedicle height), transverse angle of the pedicle, and sagittal angle of the pedicle. Statistical analysis was performed using Statistical Package for the Social Sciences (SPSS) system version 25 (Chicago, IL: SPSS Inc.).

Results: The broadest external transverse diameter was at the L5 level, with a mean of (17.54±1.6 mm) in the lumbar vertebrae. The broadest external sagittal pedicle diameter was at the L1 level (13.7±0.88 mm). The maximum transverse angle of the pedicle was at L5 with a mean of 25.39±3.10°. The maximum sagittal angle was at L1 with a mean of 5.44±0.71°.

Conclusion: The increased concern regarding the internal fixation of the spine with pedicle screw systems created the need to have almost accurate anatomical knowledge of lumbar pedicles. Due to the dynamic nature of the lumbar spine and the body's load, maximum degeneration occurs at this spine segment, making it the most commonly operated region of the vertebral column. In our study, pedicle dimensions are comparable to populations of other Asian countries. However, the pedicle dimension of our population is lower than the White American population. This morphological variation of pedicle anatomy will help surgeons choose appropriate size screws and optimum angulations to insert the implant, decreasing complications.

## Introduction

Transpedicular approaches are widely used in multiple procedures [1]; thus, the knowledge of lumbar morphometry becomes vital not only for understanding the biomechanics of the lumbar spine but also for the various interventions, such as bone biopsy, bone grafting, pedicle screw fixation, vertebroplasty, and kyphoplasty [2]. The pedicular fixation of the lumbar spine with screws is becoming popular as it is the only fixation strategy that engages all three columns. The lumbar vertebrae are the largest vertebrae of the vertebral column. They support the maximum body weight and the load of the body [3]. The pedicle has been described as "the force nucleus" of the spine, where the posterior elements converge before their communication with the more anterior vertebral body. It is the most substantial part of the vertebral body. Accurate anatomical descriptions of the shape and orientation of lumbar vertebrae are necessary for developing and using implantable devices and spinal instrumentation. The detail of pedicle morphology becomes vital as it helps in selecting the most suited pedicle screw as the dimensions of pedicles change at each vertebral level and vary according to the sex and age of patients. Information on these variations might help to prevent the failure of fixation [4]. Intensive and intimate knowledge of vertebral anatomy is necessary to prevent the complications like pedicle penetration, pedicle fracture, neurological irritation, and the leakage of cerebrospinal fluid.

Most previous studies were conducted on the Caucasian population, with minimal data available on other races. The racial variations in the skeleton are well known; hence, the morphometry of the pedicle may vary in different populations. Even within the same population, anatomical variations have been reported on the pedicle shape, size, and angulation [5]. As racial variations in pedicle anatomy have been described, this study was conducted to record the morphological dimensions of pedicles in the Central Indian population so that safe, appropriate sizes of pedicular implants can be selected.

## Materials and methods

**Figure 1:**
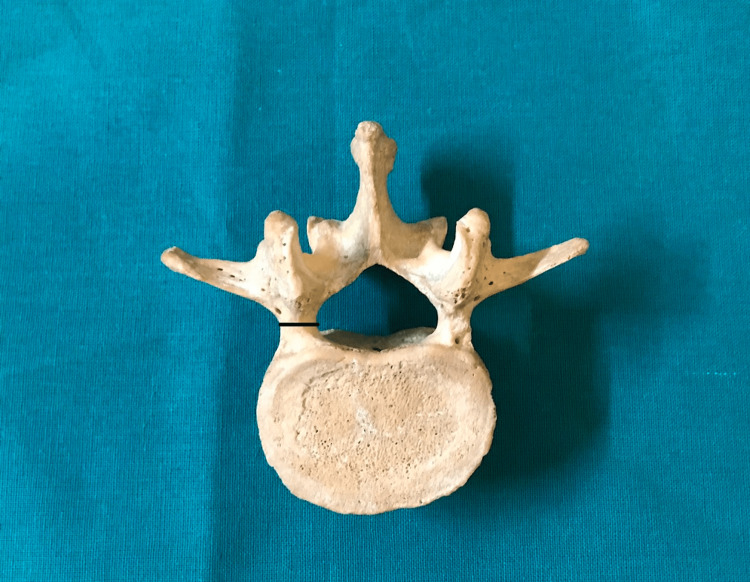
The pedicle transverse external diameter (pedicle width).

**Figure 2:**
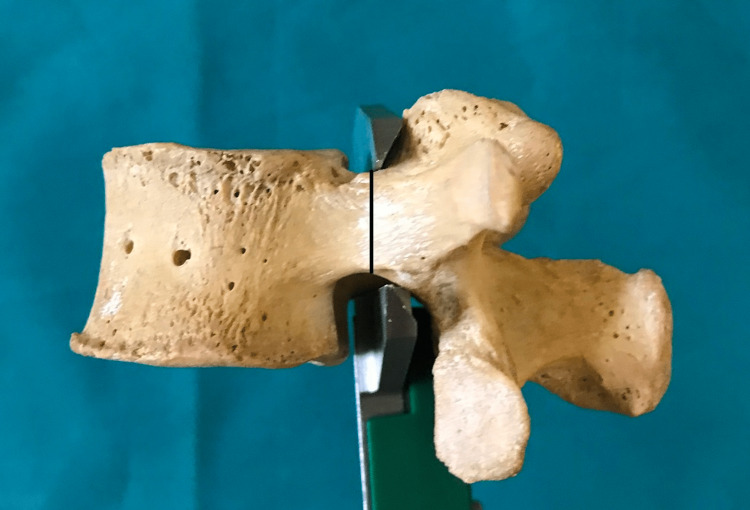
The pedicle sagittal external diameter (pedicle height).

**Figure 3:**
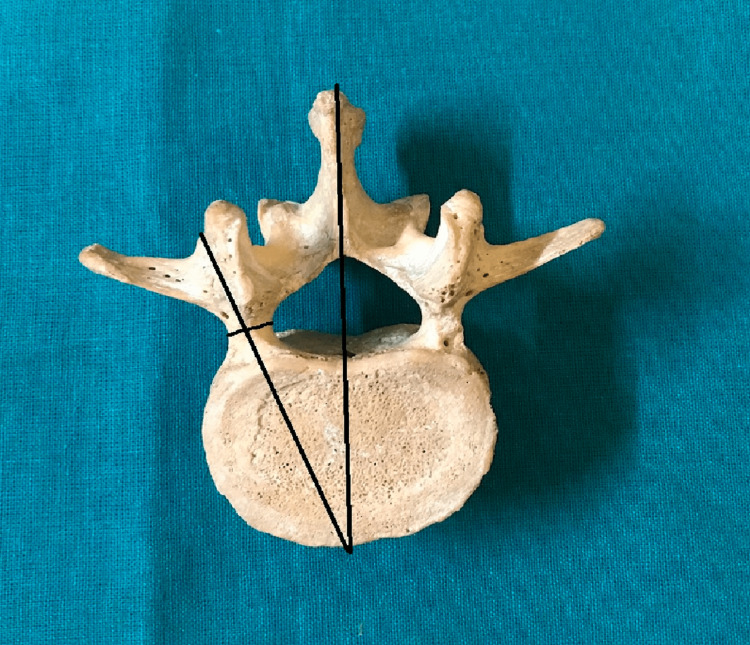
Transverse pedicle angle and pedicle width.

**Figure 4:**
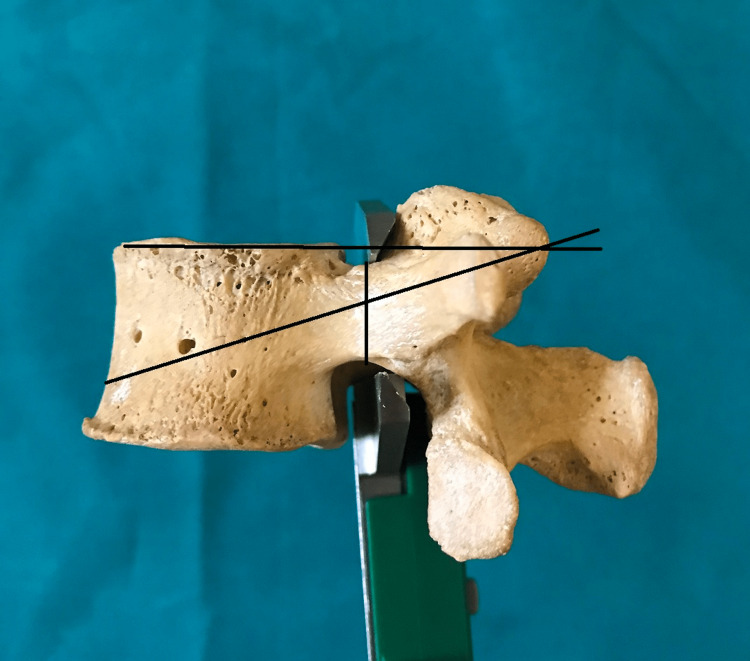
Sagittal angle and pedicle height.

The present study was conducted in a tertiary-level hospital and medical college on dry lumbar specimens available in the department of anatomy. The study's protocol was reviewed and approved by the institutional human ethics committee (#IHEC-LOP/2018/IM0206). The morphometric measurements concerning the dimensions of lumbar pedicles were performed in 20 dry lumbar vertebrae specimens. Out of these 20 dry lumbar vertebrae specimens male:female ratio is 1.5:1. The measurements were performed by two doctors, and the mean value is considered for the final assessment using male: female ratio is 1.5:Vernier calipers and a standard goniometer. The morphological measurements which were performed include the pedicle transverse external diameter (pedicle width) (Figure 1), the pedicle sagittal (pedicle height) external diameter (Figure 2), the transverse pedicle angle (Figure 3), and the sagittal angles of the pedicle (Figure 4). Pedicle transverse external diameter is a pedicle width at the narrowest diameter of the pedicle. Pedicle sagittal external diameter is a pedicle height at the narrowest pedicle diameter. The transverse angle of pedicles is defined as the angle between the pedicle axis and a line parallel to the vertebral midline measured in the transverse plane. The sagittal angle of the pedicle is defined as the angle between the pedicle axis and the superior border of the vertebral body in the sagittal plane. Statistical analysis was performed using SPSS version 25 (Chicago, IL: SPSS Inc.).

## Results

Pedicle transverse external diameter (pedicle width)

The pedicle transverse external diameter (pedicle width) varied between individuals and between levels, however, these dimensions did not differ significantly between the right and left sides. The pedicle width of the lumbar segment decreased dramatically from L1 to L5. The widest transverse external diameter of the pedicle was at L5 (17.54±1.6 mm), whereas the minimum transverse diameter was at L1 (6.66±1.13 mm).

Pedicle sagittal external diameter (pedicle height)

The narrowest pedicle sagittal external diameter (pedicle height) was observed at L5 (12.49±1.43 mm), which progressively decreased from L1 (13.7±0.88 mm). The values of pedicle sagittal external diameter (pedicle height) vary between individuals and at different levels but no statistical difference between the left and right sides.

The transverse and sagittal angles of pedicle

It is observed that there is a constant increase in the transverse angles of pedicles from L1 to L5. The most significant angle is seen at L5 (25.39±3.10°) and shallowest at L1 (8.35±2.04°). It is observed that L1 has the largest sagittal angle (5.44±0.71°) and the smallest angle at L5 (2.85±0.50°).

## Discussion

Pedicles of the lumbar vertebrae are short and robust, which plays an essential role in the transfer of weight from the neural arch to the vertebral body. Spinal pedicle fixation with screws is a commonly performed procedure for fracture fixation, post-tumor resections, and spondylolisthesis [6]. Anatomic variation in pedicle dimensions in different populations makes screw placement challenging. Previous studies have demonstrated that even with experienced surgeons, pedicle wall violations can occur in up to 29% of cases [7]. Although neurological deficits related to screw misplacement are less common, it weakens the biomechanical construct. Intimate knowledge of lumbar pedicle anatomy is imperative to decide the size of the screws and their path of insertion to prevent surgical failure or neurovascular complications [8]. In this study, different pedicular anatomical parameters were compared with measurements in previous studies of different regions and countries to sort out the crucial differences concerning Central Indian population.

Pedicle transverse external diameter (pedicle width)

The Pedicle transverse external diameter (pedicle width) gradually increases from L1 to L5 in all the studies (Table 1). The values in all Asian populations, which include India [9], Pakistan [10], Nepal [11], China [12], Korea [13], Iran [14], and Turkey [15] are lower than the White population of the United States [16] and comparable to Egypt [17] and African population [18].

**Table 1:** Pedicle width (in mm) of various regions as reported by different studies.

Study	Place	L1	L2	L3	L4	L5
Olsewski et al. [16]	USA	9.5	9.6	11.7	14.7	21.1
Tall et al. [18]	South Africa	6.9	7.6	9.6	11.9	15.1
Maaly et al. [17]	Egypt	6.8	8.8	10.1	12.9	18.9
Güleç et al. [15]	Turkey	8.21	8.55	10.54	12.67	16.57
Lotfinia et al. [14]	Iran	9.2	9.4	11.6	14.2	17.2
Kim et al. [13]	Korea	7.0	7.5	9.9	12.7	18.9
Hou et al. [12]	China	7.2	7.6	9.4	10.8	12.8
Alam et al. [10]	Pakistan	6.1	6.6	8.1	10.2	13.0
Marasini et al. [11]	Nepal	7.17	7.62	9.5	10.57	11.3
Singel et al. [19]	India (Gujrat)	8.2	8.5	10.4	13.5	18.2
Mitra et al. [9]	India (Maharashtra)	7.05	7.85	9.01	11.6	16.99
Avuthu et al. [20]	India (South India)	6.78	7.25	8.33	10.27	12.74
Acharya et al. [5]	India (North India)	7.20	7.62	8.97	11.12	13.91
This study	India (Central India)	6.66	7.37	9.41	12.21	17.54

The widest transverse external diameter of the lumbar pedicle is at the L5, 17.54 mm, compared with 21.1 mm for the White population. The values for the Korean [13] and Chinese [12] populations at L5 are 18.9 and 12.8 mm, respectively. At L1, the mean pedicle width was observed as 6.66 mm, similar to other Asian populations but lower than Egypt [17] and Africans [18], and the American population [16]. Among the Indian population, Central India has lower pedicle width than South India [18], Maharashtra [9], and Gujarat [19].

Pedicle sagittal external diameter (pedicle height)

The pedicle sagittal external diameter (pedicle height) findings are mentioned in Table 2. The pedicle height values gradually decreased from L1 to L5 and showed similar trends in all the studies compared. However, the values for American populations are greater at all levels than those with Asian populations except at L5, where the Chinese population has a higher value than other populations (20.5 mm) [12]. The sagittal diameter in the current study closely resembled that in the Pakistan study [10]. Among the Indian population, Central India has a lower pedicle height than Maharashtra [9] and Gujarat populations [19]. The pedicle height carries little significance in selecting the appropriate diameter pedicle screw as the values are higher than pedicle width at all lumbar levels.

**Table 2:** Pedicle height (in mm) of various regions as reported by different studies.

Study	Place	L1	L2	L3	L4	L5
Olsewski et al. [16]	USA	17.0	16.0	16.0	16.4	17.4
Güleç et al. [15]	Turkey	16.68	16.02	15.75	14.9	12.75
Hou et al. [12]	China	15.9	15.4	15.3	15.3	20.5
Alam et al. [10]	Pakistan	13.5	13.4	12.03	12.03	11.53
Marasini et al. [11]	Nepal	15.0	15.28	15.21	13.44	12.59
Singel et al. [19]	India (Gujrat)	14.7	15.0	14.7	14.0	13.4
Mitra et al. [9]	India (Maharashtra)	15.68	15.27	15.03	14.79	15.67
This study	India (Central India)	13.7	13.9	14.0	12.61	12.49

The transverse angle of the pedicle

The transverse angle of the pedicle findings is shown in Table 3. The trend of gradual increase from L1 to L5 observed in the current study is in accordance with the other studies. However, the values are higher in the current study, compared to American populations but lower than African populations. It suggests that pedicles are more converging in the Asian and African populations. The authors noted that Asian and African pedicles have a larger transverse angle of pedicles from L1 to L5 than the American population. In India, the Central Indian population has values comparable to Maharashtra's [9] population but higher than the South Indian population [20].

**Table 3:** Transverse pedicle angle (degree) of various regions as reported by different studies.

Study	Place	L1	L2	L3	L4	L5
Olsewski et al. [16]	USA	6.0	6.0	7.0	11.0	22.0
Tall et al. [18]	South Africa	16.3	18.2	20.8	23.5	29.1
Maaly et al. [17]	Egypt	15.0	19.0	20.0	24.0	30.0
Güleç et al. [15]	Turkey	13.08	13.84	14.33	15.69	18.98
Lotfinia et al. [14]	Iran	16.6	17.04	20.24	20.8	23.68
Alam et al. [10]	Pakistan	13.11	13.86	16.15	16.15	22.47
Mitra et al. [9]	India (Maharashtra)	9.0	10.05	12.33	14.72	29.33
Avuthu et al. [20]	India (South India)	5.38	7.15	9.7	13.37	20.4
Acharya et al. [5]	India (North India)	10.90	12.12	15.40	18.37	24.75
This study	India (Central India)	8.35	11.66	13.99	19.74	25.39

Sagittal angle of pedicle

The sagittal angle of the pedicle findings is shown in Table 4. The current findings in this context closely resembled those in Pakistan's study [10]. Wide variation in angulations at L5 demands special care at inserting the screw.

**Table 4:** Sagittal pedicle angle (degree) of various regions as reported by different studies.

Study	Place	L1	L2	L3	L4	L5
Olsewski et al. [16]	USA	5.0	6.0	6.0	6.0	5.0
Alam et al. [10]	Pakistan	3.7	3.95	4.68	4.68	4.06
Marasini et al. [11]	Nepal	17.83	15.7	15.91	13.94	12.97
Mitra et al. [9]	India (Maharashtra)	8.4	9.3	9.4	10.4	6.7
This study	India (Central India)	5.44	5.11	4.58	3.55	2.85

There has been an increased concern regarding the internal fixation of the spine with pedicle screw systems. It created the need to have almost accurate anatomical knowledge of lumbar pedicles. Due to the dynamic nature of the lumbar spine and the body load, maximum degeneration occurs at this spine segment, making it the most commonly operated region of the vertebral column.

In the present study, pedicle dimensions are comparable to populations of other regions of India (South, North, West) and other Asian populations. However, the pedicle dimension of our population is lower than the American population. This morphological variation of pedicle anatomy will help surgeons choose appropriate size screws and optimum angulations to insert the implant, which will decrease the complications. The limitation of our study is that it includes only 20 dry lumbar spine specimens from a minimal geographical area.

## Conclusions

In our study, the pedicle dimensions are measured over the dry lumbar vertebrae specimens in the central Indian population and compared to various other populations worldwide. The morphological variations in the pedicle anatomy are noted which will help the surgeons in choosing the appropriate size screws and optimum angulations to insert the implant, decreasing the complications.
